# Contrasting neonatal brain morphometry and its impact on neurodevelopmental outcome between preterm birth and congenital heart disease

**DOI:** 10.1162/IMAG.a.1063

**Published:** 2025-12-17

**Authors:** Siân Wilson, Barat Gal-Er, Daniel Cromb, Alexandra F. Bonthrone, Andrew Chew, Kuberan Pushparajah, John Simpson, Shona Falconer, Joseph V. Hajnal, Tomoki Arichi, Mary Rutherford, Chiara Nosarti, A. David Edwards, Jonathan O’Muircheartaigh, Serena J. Counsell

**Affiliations:** Research Department of Early Life Imaging, School of Biomedical Engineering & Imaging Sciences, King’s College London, London, United Kingdom; Fetal-Neonatal Neuroimaging & Developmental Science Centre, Boston Children’s Hospital, Boston, MA, United States; Division of Newborn Medicine, Boston Children’s Hospital, Boston, MA, United States; Department of Pediatrics, Harvard Medical School, Boston, MA, United States; Department of Cardiovascular Imaging, King’s College London, London, United Kingdom; Department of Fetal and Paediatric Cardiology, Evelina London Children’s Hospital, London, United Kingdom; MRC Centre for Neurodevelopmental Disorders, King’s College London, London, United Kingdom; Division of Psychological Medicine, Institute of Psychiatry, King’s College London, London, United Kingdom; Department of Forensic and Neurodevelopmental Sciences, Institute of Psychiatry, Psychology and Neuroscience, King’s College London, London, United Kingdom

**Keywords:** congenital heart disease, prematurity, MRI, neurodevelopment, morphometry

## Abstract

Congenital heart disease (CHD) and prematurity are leading causes of infant mortality and morbidity. Both groups of infants share certain common neurological sequalae, such as increased risk of neonatal brain injury and neurodevelopmental impairments later in life, leading to hypotheses that there may be shared underlying structural differences in the infant brain. However, there is no empirical evidence to support this, and our objective with this study was to compare and contrast neonatal brain structure between at-risk infant groups, then analyse their relationship with neurodevelopmental outcome. We carried out a retrospective, longitudinal, case–control analysis of 602 T2-weighted infant brain MRIs, acquired between 37 and 44 weeks postmenstrual age (PMA). The cohort comprised early preterm (n = 60), late preterm (n = 67), infants with congenital heart disease (CHD; n = 116), and term-born controls from the Developing Human Connectome Project (n = 360). We analyse structural covariance networks, a data-driven extraction of co-maturing neuroanatomical structures, capturing the variation in brain morphometry for each infant. We found distinct structural profiles in CHD and preterm infants, with minimal overlap observed between groups. A subset (n = 428) returned for neurodevelopmental follow-up at 18–24 months, and we explored the association with Bayley-III cognitive and motor scores. We found that variation in neonatal brain morphology is a significant predictor of toddler neurodevelopment in preterm infants and controls, but this association is absent in CHD. These findings suggest divergent neurobiological pathways may underlie adverse outcomes in these high-risk infants.

## 1 Introduction

Children born preterm and those born with congenital heart disease (CHD) are at increased risk of neurodevelopmental impairments across cognitive, motor, and behavioural domains (Bellinger et al., 2003; Johnson & Marlow, 2017; Latal, 2016; Marino et al., 2012). Despite a superficial similarity in neurodevelopmental risk, the underlying pathophysiology is likely to differ. Infants diagnosed with serious or critical CHD prior to corrective surgery experience a prolonged period of reduced cerebral substrate and oxygen delivery in utero and during the neonatal period (Lauridsen et al., 2017; Lim et al., 2016). This chronic hypoxia has been associated with smaller regional brain volumes and altered white matter and cortical development (Bonthrone et al., 2021; Cromb et al., 2024; Limperopoulos et al., 2010; Miller et al., 2007; Ng et al., 2020; C. Ortinau et al., 2013; Sun et al., 2015; von Rhein et al., 2015). In contrast, infants born preterm have experienced prolonged exposure to the extra-uterine environment and face multiple risk factors, such as respiratory disease, infection, poor nutrition, stress, and acute/chronic hypoxia during their neonatal clinical course (Volpe, 2009). Together, these exposures can interfere with the critical neurodevelopmental processes of the third trimester, leading to the “encephalopathy of prematurity” (Volpe, 2009, 2019) characterised by diffuse white matter injury, altered cortical development, reduced myelination, and disrupted structural connectivity (Ball et al., 2012, 2013; Batalle et al., 2017; Volpe, 2009, 2019).

Both groups of infants exhibit similar neuroimaging findings, including high prevalence of white matter injury (WMI) (Guo et al., 2019; Morton et al., 2015; Tusor et al., 2017), altered brain structure, and altered functional connectivity compared with typically developing infants (Ball et al., 2012, 2013; Batalle et al., 2017; Boardman et al., 2006; Miller et al., 2007; Ng et al., 2020; von Rhein et al., 2015). These structural and functional brain impairments are also correlated with adverse neurodevelopmental outcomes (Bonthrone et al., 2021; Claessens et al., 2018; Duerden et al., 2023; Guo et al., 2019; Kline et al., 2020; Loh et al., 2017; Peterson et al., 2000; Selvanathan et al., 2024; Vanes et al., 2021). Although these parallels suggest possible shared vulnerabilities in early brain development, it remains uncertain whether they arise from common or distinct mechanisms. To date, only one study has directly compared the brains of preterm infants with those with CHD, in which we reported differences in cortical scaling between these populations (Bonthrone et al., 2025). A broader, data-driven comparison may, therefore, help clarify whether the similar clinical outcomes observed in these groups reflect shared or divergent neurobiological pathways. Identifying points of convergence between these groups may reveal important substrates of shared vulnerability and suggest common structural anomalies. Conversely, a divergence between them may indicate that distinct pathological mechanisms culminate in white matter injury at birth and result in overlapping long-term neurodevelopmental impairments.

We aimed to address this gap in understanding by studying brain structure in four groups of infants (total n = 602) at term-equivalent age: healthy controls born at term, infants diagnosed with CHD, early preterm, and late preterm infants. The late preterm group (32.14–36.86 weeks gestational age (GA) at birth) was included because they are an understudied population; while their brain development is less affected than that of early preterm infants (<32 weeks’ gestation) (Haynes et al., 2013; Munakata et al., 2013; Vohr, 2013), their risk of neurodevelopmental anomalies remains higher than that of term-born peers (Cheong et al., 2017; Woythaler et al., 2011). We, therefore, hypothesised that in our analysis, they would represent an intermediate point between the early preterm and control infants, and an entirely distinct group from the CHD infants, providing a continuum across which to assess the sensitivity of our analyses for detecting subtle structural differences between groups.

To study structural brain development across these groups, we used a whole-brain, data-driven approach that does not rely on predefined regions of interest. For this task, we applied independent component analysis (ICA), an unsupervised learning method routinely applied to fMRI data (Comon, 1994), but in this case applied to T2-weighted MRI to explore the spatial domain, extracting structural covariance networks (SCNs) that represent the variation in relative volumes and morphometry of brain structures across a population (Avants & Gee, 2004; Studholme et al., 2001; Thompson et al., 2000; Wilson et al., 2024). In the context of our mixed-patient cohort, SCNs highlight the coordinated altered structural maturation of specific brain regions (Bassett et al., 2008; Fenchel et al., 2020; Heinze et al., 2015; Li et al., 2024; Vanes et al., 2021), and allow us to identify the cerebral morphological alterations that typify the neonatal brain in CHD, early preterm and late preterm infants. By correlating the weightings of each network, we sought to determine whether vulnerability to adverse neurodevelopmental outcomes arises from shared or distinct structural foundations in these high-risk populations.

## 2 Materials and Methods

### 2.1 Ethical approval

We used the open-access developing Human Connectome Project (Edwards et al., 2022) neonatal MRI database (www.developingconnectome.org, https://nda.nih.gov/edit_collection.html?id=3955) to retrospectively obtain brain MRI data of term-born controls, and early and late preterm infants. Brain MRI scans for the CHD group were acquired as part of The Congenital Heart Disease Imaging Programme (CHIP). Research ethical approval was obtained (dHCP: 14/LO/1169; CHIP: 07/H0707/105 and 21/WA/0075) and informed written consent was obtained from parents prior to scanning of the infants.

### 2.2 Recruitment

Inclusion criteria were infants delivered up to 42 weeks GA, who underwent an MRI scan between 37 and 44 weeks post-menstrual age (PMA). The exclusion criteria for each group are tabularised in the Supplementary Table S1.

#### 2.2.1 Term control infants

Exclusion criteria were GA at birth <37 weeks, admission to the neonatal intensive care unit, a 1^st^ degree relative with a diagnosed neurodevelopmental disorder, cognitive, language, or motor composite scores below 70 (<2 standard deviations from the test mean) at 18 months, assessed using the Bayley-III scales of infant and toddler neurodevelopment (Bayley, 2012).

#### 2.2.2 CHD infants

Inclusion criteria were infants diagnosed with serious or critical CHD (Ewer et al., 2011), who were expected to require cardiac catheterisation or surgery within 1 year and who underwent pre-operative neonatal neuroimaging (Table 1). These infants were recruited from the neonatal intensive care unit at St Thomas’ Hospital, London. Critical CHD was defined as infants with hypoplastic left heart syndrome, hypoplastic right heart syndrome, interrupted aortic arch, pulmonary atresia with an intact ventricular septum, and simple transposition of the great arteries; and all infants requiring surgery or cardiac catheterization within the first 28 days after birth with any of the following: aortic valve stenosis, coarctation of the aorta, pulmonary valve stenosis, pulmonary atresia with ventricular septal defect, tetralogy of Fallot, and total anomalous pulmonary venous drainage. Serious CHD was defined as any cardiac lesion not defined as critical, requiring cardiac catheterisation or surgery between 1 month and 1 year of age. Exclusion criteria were birth <37 weeks GA, suspected or confirmed chromosomal abnormality/congenital syndrome, previous neonatal surgery (excluding cardiac catheterisation procedures), or suspected congenital infection.

**Table 1.** Participant demographics and CHD cohort characteristics.

(a)				
All participant demographics				
Characteristic	Control (term) (n = 360)	CHD (n = 116)	Early preterm (n = 60)	Late preterm (n = 67)
GA at birth, weeks (range; median)	37.0–42.71(40.14)	37.0–41.57(38.5)	23.0–31.86(28.71)	32.14–36.86(34.86)
PMA at scan, weeks (range; median)	37.43–44.71(41.43)	37.14–42.43(39.14)	38.29–44.86(41.36)	37.0–45.14(40.86)
Sex, male (n),	188	63	33	39
multiple birth, twins (n)	16	6	18	25
IMD (median; IQR)	-1010.60 (-6903.1–3837.9)	2829.1 (-5280.1–10982.4)	1436.77 (-5277.10–6007.90)	-
Birthweight z-score (range; median)	-3.45–2.26 (-0.19)	-3.10–1.89 (-0.18)	-2.75–1.01 (-0.41)	-2.98–3.10 (-0.12)

(b)				
CHD cohort characteristics				
Cardiac group	Diagnosis		Count (n)	
Group 1—abnormal streaming of flow (n = 54)	Transposition of the great arteries (TGA)		45	
	Atrioventricular septal defect (AVSD)		1	
	Double outlet right ventricle (DORV)		3	
	Total anomalous pulmonary venous drainage (TAPVD)		1	
	Truncus arteriosis		4	
Group 2—left-sided heart lesions (n = 34)	Aortic stenosis		3	
	coarctation of the aorta		22	
	Hypoplastic left heart syndrome (HLHS)		7	
	Hypoplastic aortic arch		1	
	Interrupted aortic arch		1	
Group 3—right-sided heart lesions (n = 28)	Hypoplastic right heart syndrome (HRHS)		1	
	Pulmonary atresia		7	
	Pulmonary stenosis		4	
	Tetralogy of Fallot		15	
	Tricuspid atresia		1	

(a) Participant demographic and birth characteristics for all infant groups. Values are presented as range (median) unless otherwise stated. Counts are indicated by (n). GA = gestational age at birth; PMA = postmenstrual age at scan; IMD = index of multiple deprivation, reported as median (IQR).

(b) Congenital heart disease (CHD) cohort characteristics and cardiac group classification. Infants were categorised according to the sequential segmental approach, based on the predominant haemodynamic physiology of the underlying defect: Group 1—Aanormal streaming of flow; Group 2—left-sided lesions; and Group 3—right-sided lesions. Counts (n) represent the number of infants with each diagnosis, arranged by group.

#### 2.2.3 Early preterm infants

Inclusion criteria were GA at birth <32 weeks.

#### 2.2.4 Late preterm infants

Inclusion criteria were GA at birth 32–36^+9^ weeks.

Additional exclusion criteria for all infants were no/motion corrupted neonatal brain MRI, and major lesions identified on neonatal MRI (e.g. arterial ischaemic stroke or parenchymal haemorrhage) after review by two perinatal neuroradiologists.

### 2.3 Neonatal MRI acquisition

All infants were scanned using the Philips Achieva 3 Tesla system (Philips Healthcare, Best NL) located in the neonatal unit at St Thomas’ Hospital, London, with the dHCP neonatal acquisition protocol (Edwards et al., 2022; Hughes et al., 2017). Participants were scanned without sedation, using a dedicated neonatal brain imaging system (Hughes et al., 2017) that includes a custom 32-channel receive neonatal head coil (Rapid Biomedical GmbH, Rimpar DE), inflatable pads for positioning and reduction of head movement (Pearltec, Zurich CH), ear putty and earmuffs for noise attenuation (President Putty, Coltene Whaledent, Mahwah, NJ, USA). To promote natural sleep and avoid the use of sedation, infants were fed, swaddled, and positioned in a vacuum jacket prior to scanning. Scans were supervised by a neonatal nurse and/or paediatrician, who monitored heart rate, oxygen saturation, and body temperature throughout image acquisition (Hughes et al., 2017).

T2-weighted MRI was acquired using a Turbo Spin-Echo (TSE) sequence, in two stacks of 2D slices (in sagittal and axial planes); slice thickness = 1.6 mm acquired with an overlap of 0.8 mm; in-plane resolution = 0.89 mm; TR = 12 s; TE = 156 ms; flip angle = 90°; field of view = 145 × 145 × 108 mm; SENSE factor 2.11 (axial), and 2.58 (sagittal). We applied in-house specialised neonatal pipelines for motion correction and super-resolution reconstruction to reconstruct volumes to 0.5 mm^3^ isotropic resolution (Cordero-Grande et al., 2019; Kuklisova-Murgasova et al., 2012). We then applied the dHCP structural pipeline for bias field correction and brain extraction (Makropoulos et al., 2018). Visual quality control by experts in perinatal MRI (A.B., S.W.) was subsequently conducted to remove subjects with residual motion artefact.

### 2.4 Cardiac grouping

The CHD cohort was further grouped according to the sequential segmental approach (R. H. Anderson et al., 1984) which standardises echocardiographic descriptions of cardiac anatomy and allows classification of congenital heart disease according to the predominant haemodynamic impact of the underlying defect. Infants were categorised into three groups reflecting the principal physiology of the circulation:

Group 1 – Abnormal streaming of blood: lesions characterised by abnormal mixing or distribution of oxygenated and deoxygenated blood.
Group 2 – Left-sided heart lesions: defects primarily resulting in obstruction of systemic outflow.
Group 3 – Right-sided heart lesions: defects primarily resulting in obstruction of pulmonary outflow.

This physiologically oriented classification of our cohort was achieved through consultation with expert paediatric cardiologists (J.S. and K.P.), and this approach has been used in previous studies to investigate how differing circulatory physiologies influence cerebral development (Bonthrone et al., 2021, 2025; Cromb et al., 2023, 2024; Kelly et al., 2017). While some heterogeneity inevitably remains within each category, grouping by predominant haemodynamic physiology provides a pragmatic and clinically meaningful framework for exploring shared effects on brain development. A comprehensive breakdown of the specific diagnoses that were assigned to each category and the distribution of infants between groups and diagnoses are shown in Table 1.

### 2.5 Neurodevelopmental assessment

Four hundred and twenty-eight infants attended a follow-up assessment and completed the Bayley Scales of Infant and Toddler Development–Third Edition (Bayley-III), administered by a developmental paediatrician, and cognitive and motor composite scores were included in this analysis (Supplementary Table S2). Although the language score was collected, we did not include this in our analysis due to the diversity of the primary language spoken at home in our study population. The median age of assessment was 18 months for the dHCP cohort (including controls and early preterm subjects), and 22.2 months for the CHD group (Supplementary Table S2). Only five late preterm subjects attended a follow-up assessment and, therefore, this group was not included in our analysis of neurodevelopmental outcome.

### 2.6 Socioeconomic status

To derive an estimate of socioeconomic status for each infant, we calculated the index of multiple deprivation (IMD) using the postcode at birth for all infants who attended the follow-up assessment. IMD measures relative deprivation in small areas across each of the constituent nations of the United Kingdom, encompassing factors such as income, employment, education, health, and crime (http://imd-by-postcode.opendatacommunities.org/).

### 2.7 MR Image registration and Jacobian determinant calculation

Each skull-stripped, brain-masked native subject T2-weighted dataset was registered to a dHCP week-specific template (according to PMA at scan) (https://gin.g-node.org/BioMedIA/dhcp-volumetric-atlas-groupwise) (Schuh et al., 2018), then to the central common space corresponding to GA of 40 weeks (Schuh et al., 2018) using Symmetric diffeomorphic registration (SyN) algorithm and cross correlation from Advanced Normalisation Tools (ANTs), version 3.0 (Avants et al., 2008). The log-Jacobian was computed on the concatenated transform, which included both brain parenchyma and CSF spaces, to capture global structural covariance patterns across tissue boundaries. The log Jacobian determinant of the warp represents the contraction and expansion of brain regions, such that higher log-Jacobian values represent brain regions that contracted in the subject-to-template transformation, while smaller values represent expanding regions in the subject-to-template transformation (Avants & Gee, 2004). The Jacobians for the entire cohort were concatenated, creating a single 4D set of volumes which were used as the input for ICA extraction of networks, described below (Varoquaux et al., 2010).

### 2.8 Independent component analysis (ICA) of Jacobian determinants

A canonical ICA algorithm was applied to the log Jacobian determinants, implemented in Python with the nilearn package (Abraham et al., 2014; Varoquaux et al., 2010). Application of ICA in the spatial domain extracts distinct networks that represent co-maturing brain structures, termed “structural covariance networks”, as described previously (Douaud et al., 2014; Llera et al., 2019; O’Muircheartaigh et al., 2014; O’Muircheartaigh & Jbabdi, 2018; Wilson et al., 2024). The extracted networks represent an “unmixing” of the signal into independent sources with non-Gaussian distributions. To determine the optimal number of SCNs, a literature review of previous work applying this technique was conducted and (*n* = 40) was chosen to balance robustness and interpretability (Eyre et al., 2021; Vanes et al., 2021). In exploratory analyses using *n* = 50–60 components, we observed finer subdivisions of neuroanatomical regions and bilateral splits of otherwise coherent structures, resulting in networks with reduced anatomical coherence (Supplementary Fig. S2).

To assess the reproducibility of the ICA decomposition, we conducted a split-half reliability analysis**.** The cohort was randomly divided into two halves (balanced for clinical group, GA at birth, and PMA at scan), and ICA (*n* = 40) was run separately on each subset. Components were visually matched across halves, and similarity was quantified using the Dice coefficient. The median Dice coefficient across matched components was 0.37 (IQR 0.18–0.56), indicating good reproducibility, consistent with prior ICA-based neuroimaging studies. In addition to split-half reliability analysis, we conducted a sensitivity analyses to demonstrate the consistency of the neuroanatomy of components between groups, extracting them exclusively within the preterm and/or CHD groups (Supplementary Figs. S3 and S4).

To quantify the representation of each network in each subject, FSL’s general linear model was applied to the component maps and the Jacobians, extracting a weighting for each network in each infant for subsequent regression analysis (M. J. Anderson & Robinson, 2001; Winkler et al., 2014).

### 2.9 Statistical analysis

**Fig. 1.**
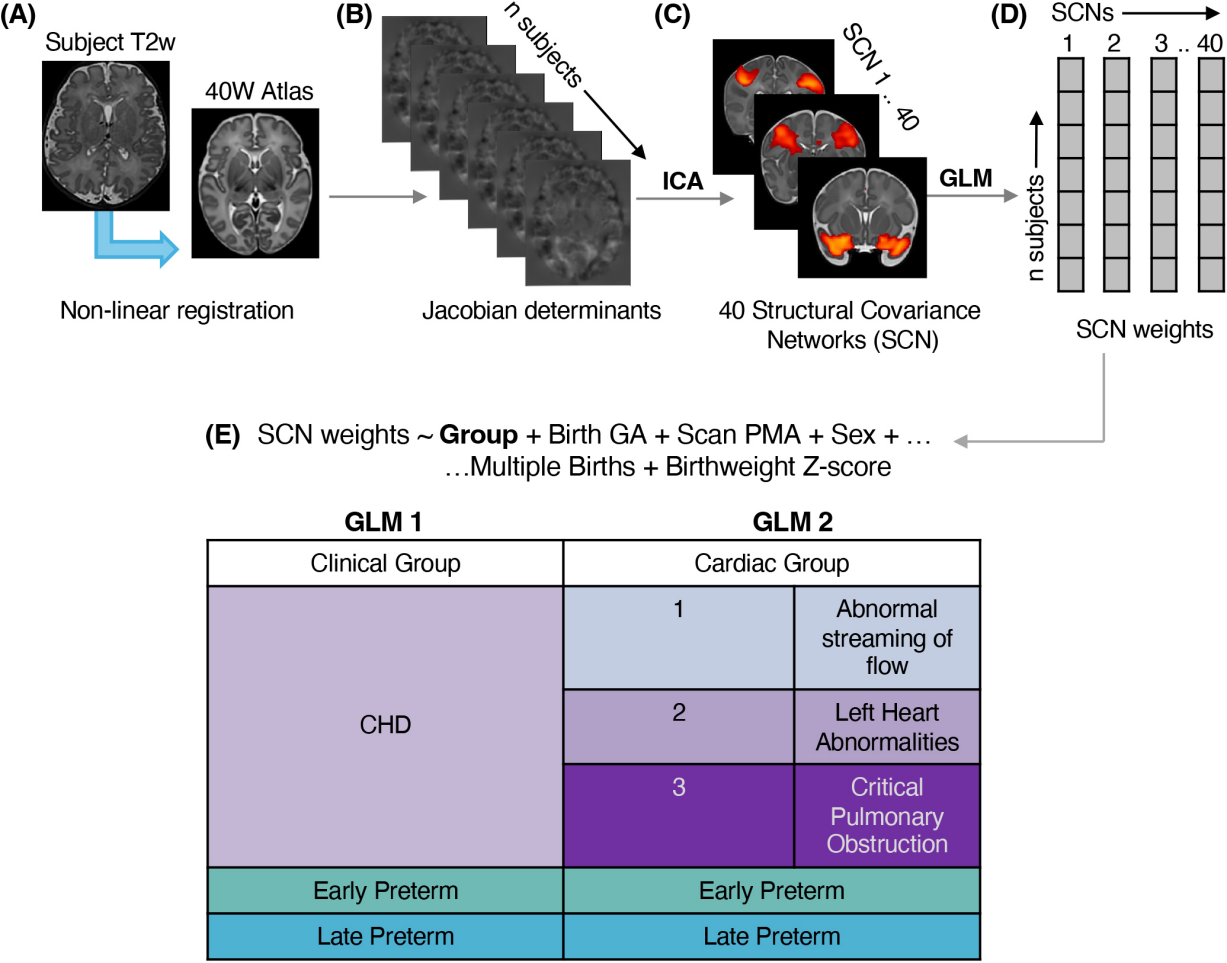
Methods pipeline. (A) T2w neonatal MRI is registered to the dHCP 40-week atlas, using non-linear registration. (B) The log Jacobian determinant of the non-linear warp is computed. (C) The canonical ICA algorithm is applied to the Jacobians of the entire cohort, to extract 40 SCNs. (D) An SCN weighting (or “modes”) are extracted for each network in each subject. (E) Two GLMs were fit to examine the sources of variance across the mixed patient cohort for each SCN, accounting for covariates such as birth GA, scan PMA, and sex, and (i) either clinical group or more granular, (ii) cardiac group.

Linear regression models were used to examine variation in network weightings (“modes”) between groups, implemented in R (v4.1.2; R Core Team 2021). The first model compared the entire CHD group with controls, early and late preterm groups, and the second compared the CHD subgroups defined above (Fig. 1). In both GLMs, gestational age at birth (GA), postmenstrual age at scan (PMA), sex, multiple birth status (singleton or twin), and birthweight Z-score were included as covariates.

These models can be expressed as:

$$\begin{array}{l} SCN\;Network\;Weighting\sim \text{ Group}+\text{GA }\left(\text{at Birth}\right)\\ \;\;\;+\text{PMA }\left(\text{at Scan}\right)+\text{Sex}+\text{Multiple Birth}\\ \;\;\;+\text{Birthweight Z}-\text{score} \end{array}$$ [1]

Then, to examine whether neonatal brain morphometry was significantly associated with neurodevelopmental outcome, we fitted GLM [2] for each group (controls, CHD, and early preterm) and each outcome domain (motor and cognitive). Again, FDR correction was applied across 40 SCNs per group and outcome. Multiple Birth was not significant in this model and was therefore removed from the final equation.

This model can be expressed as:

$$\begin{array}{l} Outcome\;Score\sim \text{ SCN Network Weighting}\\ \;\;\;+\text{GA }\left(\text{at Birth}\right)+\text{PMA }\left(\text{at Scan}\right)+\text{Sex}+\text{IMD} \end{array}$$ [2]

For both models [1] and [2], results were corrected for multiple comparisons across 40 models (one per SCN) using false discovery rate (FDR)–adjusted *p*-values (q < 0.05).

## 3 Results

### 3.1 Participants

Demographic and birth characteristics of the participants are shown in Table 1. For the CHD cohort, the primary CHD diagnoses and number of subjects in each cardiac group are summarized in the lower half of Table 1.

### 3.2 Structural covariance networks

**Fig. 2.**
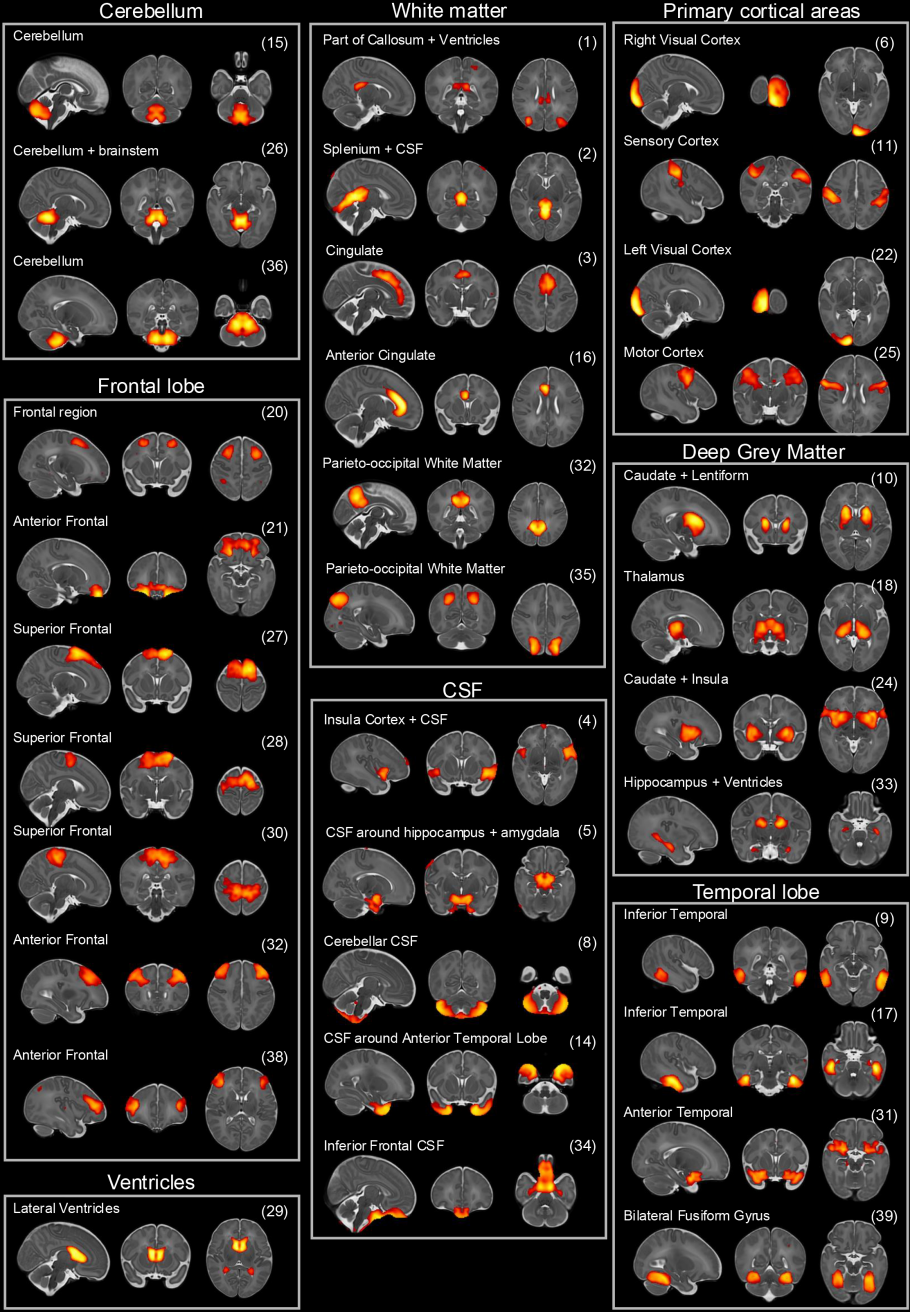
Structural covariance networks grouped by neuroanatomy. Each SCN has an anatomical label and a reference number (1–40).

We applied ICA using the Jacobians of the entire cohort to extract 40 SCNs (Fig. 1). Collectively these SCNs encompassed every tissue type and represented anatomically meaningful structures in neonatal brain development (Fig. 2). The networks included white matter structures such as the cingulate and corpus callosum, cerebellar regions, deep grey matter structures such as the thalamus and hippocampus, and specific cortical areas such as the somatosensory and motor cortex. Networks were often bilateral and symmetric between left and right hemispheres. The networks were sub-grouped to qualitatively explore their distribution, highlighting a large proportion of networks within the frontal and temporal lobes (Fig. 2).

### 3.2 Morphometric differences between CHD and preterm infants

**Fig. 3.**
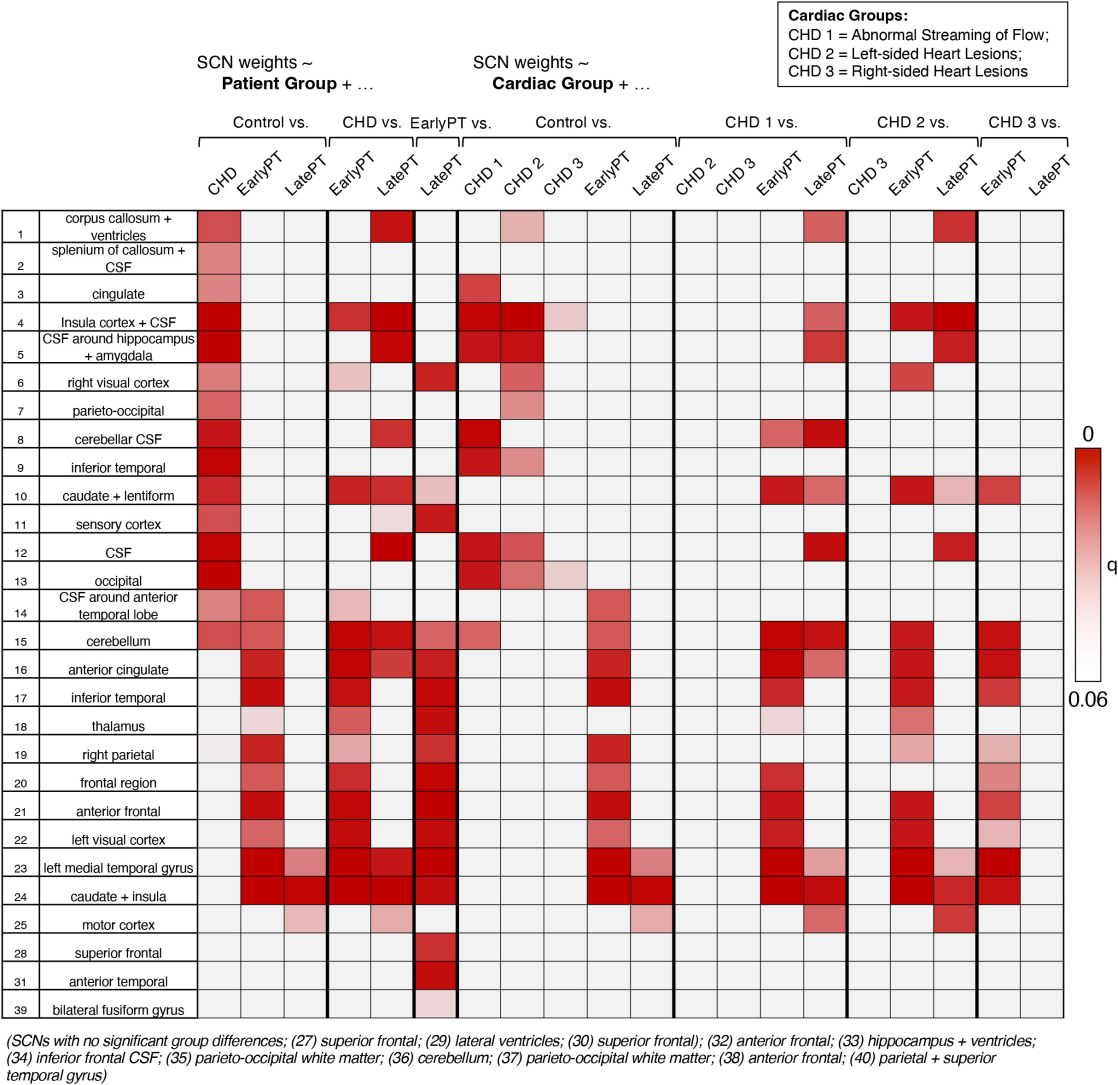
The significant effect of CHD, early preterm and late preterm birth on SCNs. Heatmap showing the significance (FDR-corrected q values) of group effects on independent component (IC) weights derived from the spatial independent component analysis (ICA) of log-Jacobian determinants. Columns represent pairwise group contrasts from the general linear models (GLMs): patient group comparisons (*Control vs.* CHD, Early Preterm, Late Preterm; *CHD vs.* Early Preterm, Late Preterm) and cardiac subgroup comparisons (CHD1–3 vs. Controls, Early Preterm, Late Preterm, and between cardiac subgroups). Rows correspond to individual SCNs, with a numerical label matched to Figure 2 (1–40), only SCNs for which there was at least one significant group effect are shown. Colour intensity reflects the statistical significance of group differences (red indicates lower q values), with lighter shading denoting less significant effects (q approaching 0.06). Black grid lines separate the main model contrasts. Cardiac subgroups were defined as CHD1 = Abnormal Streaming of Flow; CHD2 = Left-Sided Heart Lesions; CHD3 = Right-Sided Heart Lesions.

After extracting individual subject weightings for each network, a GLM was fit to test for differences between each patient group in each network (Fig. 1). The results are summarised in Figure 3, which highlights the significant networks (q < 0.05) for each groupwise comparison after FDR correction. Fifteen SCNs were identified where CHD was a significant predictor of variance (Fig. 3), 13 of which were significant for CHD alone but not different between early/late preterm and controls. These included the cingulate, caudate, lentiform, and corpus callosum networks. Similarly, for the early preterm group, 11 significant SCNs were identified, 8 of which were uniquely different, encompassing 3 frontal lobe networks, 2 temporal lobe networks and bilateral thalami. For the late preterm group, only one network was significantly different, encompassing the primary motor cortex. There were 16 networks that were significantly different between early and late preterm cohorts, 10 of which were also different between early preterm and controls. Overall, there was minimal overlap in the significant networks between patient groups, with each group showing distinct networks when compared with typically developing controls.

**Table 2.** Neonatal SCNs that are significant predictors of cognitive and motor outcome score in toddlerhood.

Bayley outcome	Group	Term	β	Std. error	Statistic	q value	Significance
Cognitive score	Controls	Lateral ventricles	-0.079	0.040	-1.990	0.047	*
		GA	-0.058	0.618	-0.094	0.925	
		PMA	1.410	0.437	3.240	0.001	**
		Male	-0.536	1.170	-0.458	0.647	
		IMD	<0.001	<0.001	2.140	0.034	*
		Anterior temporal	0.096	0.048	2.000	0.047	*
		GA	0.257	0.603	0.427	0.670	
		PMA	1.280	0.431	2.970	0.003	**
		Male	-0.560	1.170	-0.479	0.632	
		IMD	<0.001	<0.001	2.250	0.025	*
		Anterior frontal	0.101	0.050	2.030	0.043	*
		GA	0.185	0.603	0.306	0.759	
		PMA	1.390	0.435	3.200	0.002	**
		Male	-0.799	1.180	-0.680	0.497	
		IMD	<0.001	<0.001	2.000	0.047	*
		Right visual cortex	0.102	0.045	2.280	0.023	*
		GA	0.214	0.601	0.355	0.723	
		PMA	1.150	0.433	2.660	0.008	**
		Male	-0.527	1.170	-0.451	0.652	
		IMD	<0.001	<0.001	2.400	0.017	*
		Medial occipital	0.066	0.033	1.990	0.047	*
		GA	0.328	0.606	0.542	0.588	
		PMA	1.190	0.433	2.740	0.007	**
		Male	-0.626	1.170	-0.535	0.593	
		IMD	<0.001	<0.001	2.200	0.029	*
	Early preterm	Cingulate	0.170	0.075	2.270	0.028	*
		GA	1.550	0.559	2.780	0.008	**
		PMA	1.900	0.622	3.050	0.004	**
		Male	-8.330	3.360	-2.480	0.017	*
		IMD	<0.001	<0.001	1.380	0.175	
		Lateral ventricles	-0.213	0.081	-2.630	0.012	*
		GA	0.835	0.584	1.430	0.160	
		PMA	2.020	0.609	3.310	0.002	**
		Male	-4.200	2.680	-1.570	0.124	
		IMD	<0.001	<0.001	1.870	0.068	
		Inferior temporal	0.171	0.078	2.190	0.034	*
		GA	1.560	0.562	2.770	0.008	**
		PMA	2.070	0.621	3.330	0.002	**
		Male	-6.490	2.980	-2.180	0.035	*
		IMD	0.000	0.000	2.230	0.031	*
		Cerebellum	0.321	0.152	2.100	0.041	*
		GA	1.290	0.560	2.300	0.026	*
		PMA	1.820	0.633	2.870	0.006	**
		Male	-3.410	2.750	-1.240	0.221	
		IMD	<0.001	<0.001	2.030	0.048	*
Motor score	Controls	Superior frontal	-0.111	0.035	-3.170	0.002	**
		GA	0.806	0.512	1.570	0.117	
		PMA	-0.220	0.415	-0.531	0.596	
		Male	-0.197	1.000	-0.197	0.844	
		IMD	<0.001	<0.001	0.531	0.596	
		Right visual cortex	0.079	0.039	2.040	0.042	*
		GA	0.869	0.517	1.680	0.094	
		PMA	0.310	0.372	0.833	0.405	
		Male	-0.536	1.000	-0.534	0.594	
		IMD	<0.001	<0.001	0.601	0.548	
		Parieto occipital	0.061	0.025	2.410	0.016	*
		GA	0.758	0.517	1.470	0.144	
		PMA	0.403	0.369	1.090	0.276	
		Male	-0.760	1.000	-0.757	0.449	
		IMD	<0.001	<0.001	0.490	0.625	
	Early preterm	Cerebellum & brainstem	0.288	0.129	2.230	0.031	*
		GA	2.710	0.639	4.240	<0.001	**
		PMA	1.980	0.668	2.960	0.005	**
		Male	-2.630	2.960	-0.890	0.378	
		IMD	<0.001	<0.001	0.042	0.967	
		Cerebellum	0.487	0.155	3.130	0.003	**
		GA	2.040	0.571	3.570	0.001	**
		PMA	1.780	0.645	2.770	0.008	
		Male	-3.060	2.800	-1.090	0.280	
		IMD	<0.001	<0.001	0.814	0.419	

Linear regression models were used to assess the association between SCN weighting and Bayley-III cognitive and motor composite scores for each group, controlling for gestational age at birth (GA), postmenstrual age at scan (PMA), sex, and index of multiple deprivation (IMD). The β coefficient represents the effect size of each association, indicating the direction and magnitude of the relationship between network weighting and outcome score. *p* values were corrected for multiple comparisons across 40 SCNs using the false discovery rate (FDR; q < 0.05). * indicates q < 0.05; ** indicates q < 0.01.

### 3.3 CHD subtype differences to early and late preterm infants

The subject weightings were then explored with a second GLM, further subdividing the CHD cohort into three cardiac subtypes (Fig. 3). The aim of this was to explore which CHD subtypes were driving the significant differences between networks, to draw a more refined comparison between CHD subtypes and the preterm brain. Eight networks were identified where the “Abnormal Streaming of Flow” Group was different from controls, three of these networks were exclusively significant for this group, which included the cerebellum, cerebellar CSF, and the cingulate (Fig. 3). With the “Left-Sided Heart Lesions” Group, there were also eight significantly different networks, and three unique to this group, including the corpus callosum and ventricles, the right visual cortex, and a parieto-occipital region. There were only two significantly different networks between the “Right-Sided Heart Lesions” Group and controls, with no networks unique to this group. No statistically significant networks distinguished between CHD subgroups.

When we stratified the CHD infants into subgroups, and compared them with the early preterm group, an identical series of networks were highlighted, matching the CHD vs. early preterm comparison. When exploring the difference between each CHD group and late preterm, there were 11 networks significantly different between Group 1 and late preterm, 8 for Group 2 vs. late preterm, and none for Group 3 vs. late preterm.

### 3.4 Follow-up assessment with the Bayley scale shows differences between at-risk infant groups and controls

The control group showed higher motor and cognitive composite scores than the early preterm and CHD groups (Motor; control mean (SD) = 102 (10.5) vs. early preterm mean (SD) = 95 (11.5) vs. CHD mean (SD) = 94 (10.6); p < 0.001, cognitive; control mean (SD) = 104 (8.8) vs. early preterm mean (SD) = 96 (10.1) vs. CHD mean (SD) = 97 (10.2), p < 0.001; Supplementary Table S2). We found no significant difference in outcome score between the CHD and early preterm groups (Supplementary Fig. S1).

### 3.5 Specific, distinct SCNs are predictors of motor and cognitive outcome in controls and early preterm infants

We fit GLMs to explore the relationship between SCN weightings and Bayley composite scores (motor and cognitive), while accounting for other factors such as age at birth, age at scan, sex, and IMD (Table 2). Multiple SCNs were significantly associated with cognitive and motor score in the control group. For cognitive score, this included anterior temporal, anterior frontal, visual cortex, lateral ventricles, and medial occipital regions. For motor score, the significant networks encompassed superior frontal, visual cortex, and parieto-occipital regions. In the control group, IMD showed a significant negative association with cognitive outcome scores in several models for the control group (Table 2), indicating that higher deprivation was linked to lower cognitive performance. No significant association between IMD and motor outcomes was observed in any group.

In the early preterm infants, the cingulate, inferior temporal, and cerebellar networks were significantly correlated with cognitive score, and the only overlap with controls was the lateral ventricles. For the motor score, the cerebellum and brainstem networks were also significant predictors in the early preterm group. There were no networks that were significantly associated with either motor or cognitive outcome for the CHD group.

The β coefficients in Table 2 represent the effect sizes for each network–outcome association. Notably, these values were generally larger in the early preterm group than in controls, suggesting a stronger relationship between brain morphometry and neurodevelopmental outcome in this cohort.

## 4 Discussion

This study represents the first direct comparison of whole brain morphometry between infants with CHD and infants born preterm. We investigated whether common brain systems are altered by CHD and prematurity at the start of life which may further explain why they have similar neurological, cognitive, and behavioural phenotypes in childhood. To address this, we analysed T2-weighted MRI data acquired at term-equivalent age to identify similarities in brain morphometry between these high-risk paediatric groups. To capture whole-brain variation, while reducing dimensionality for interpretability, we applied ICA in the spatial domain to Jacobian determinants. We extracted SCNs that represent the drivers of variance within the input data, representing co-maturing regions relevant to the specific mixed-patient cohort of neonates, including preterm infants and severe/critical CHD.

Although infants with CHD are at increased risk of being born preterm, we restricted the CHD cohort to those delivered at ≥37 weeks’ gestation. This design choice allowed prematurity and CHD to be examined as independent influences on early brain development. Consequently, the findings reported here reflect structural differences attributable to CHD itself, rather than the combined effects of CHD and prematurity. Future studies including preterm CHD populations will be important to explore how these risk factors interact to influence neurodevelopment.

Our findings revealed minimal overlap in brain morphometric alterations between the CHD and preterm groups, with few shared differences relative to controls. Of the 40 structural covariance networks analysed, many differed between individual patient groups and controls, yet only 2 were significantly different from controls across more than 1 high-risk group. A similarly large number of networks also differed between the high-risk groups themselves. Together, these results imply distinct neonatal brain phenotypes in CHD, early preterm and late preterm infants.

Furthermore, when we examined whether SCNs were significant predictors of neurodevelopmental outcome score in toddlerhood, we found that distinct networks were associated with outcome score for early preterm and control infants, with a larger effect size in the early preterm group, however, we found no association between brain morphometry in the CHD group. Our findings suggest that the shared vulnerability to longer-term neurodevelopmental delay is unlikely to be underpinned by the same structural foundations in these at-risk groups of neonates.

A larger proportion of networks were distinct for the CHD infant group than for any other patient group, followed by early preterm infants. This suggests that CHD infants experience the most severe alterations in brain morphometry among the groups studied. As expected, the late preterm group represented an intermediate point between early preterm infants and controls, with only three significantly different networks compared with controls but many distinct networks when compared with the early preterm and CHD groups. This finding suggests that, despite known differences in brain structure and function (Munakata et al., 2013; Rogers et al., 2014) and the greater burden of neurodevelopmental disability observed in late preterm infants compared with term-born infants (Cheong et al., 2017; Vohr, 2013), the brain morphometry of late preterm infants more closely resembles that of typically developing infants than that of early preterm or CHD infants.

Previous work in preterm and CHD neonates has found evidence of structural brain abnormalities at birth which have important consequences later in life, explaining some of the variance in future neurodevelopmental outcome. Neuroimaging studies in the preterm population have identified reduced cerebellar, thalamic, and cortical volumes (Boardman et al., 2006; Dimitrova et al., 2021; Inder et al., 2005; Peterson et al., 2000; Tam et al., 2009), altered white matter development (Back, 2014), impaired thalamocortical connectivity (Ball et al., 2012, 2013), and reduced gyrification (Papini et al., 2020). Our analysis reinforces these findings, observing unique bilateral thalami, caudate and insula, frontal and temporal lobe morphometry in the early preterm group, and a significant association between the cerebellar and brainstem networks and motor outcome score. Interestingly, the SCNs we identified showed a high degree of spatial correspondence with neonatal resting-state functional connectivity networks (Eyre et al., 2021), suggesting that future studies might benefit from directly comparing functional connectivity between CHD and preterm infants, to further elucidate the origins of neurodevelopmental risk in these patient groups.

In contrast to the early preterm group, the CHD group was characterised by distinct morphometry in CSF spaces, corpus callosum networks, and other deep grey matter structures, such as the caudate/lentiform nuclei. The distinct morphometry of CSF spaces in the CHD group has been highlighted by previous work (Ng et al., 2020), and may reflect the altered sulcal patterns and reduced surface area expansion recently reported in fetuses with CHD (C. M. Ortinau et al., 2019). The vulnerability of the deep grey matter was also highlighted by our previous work showing a pre- to postoperative relationship between longer cardiopulmonary bypass duration, higher preoperative creatinine levels, and impaired growth in the brainstem and caudate nuclei (Cromb et al., 2023). This may be due to the basal ganglia having relatively high metabolic demands during infancy, rendering them particularly susceptible to acute hypoxia/ischaemia (Back, 2014; Rocha-Ferreira & Hristova, 2016). We did not detect any significant association between neonatal brain morphometry and outcome in the CHD group, which suggests that other brain or cardiac features may be more significant drivers of variance in their neurodevelopmental phenotype.

Several factors may explain the differences in structural brain morphometry between high-risk groups. While both groups may be exposed to a hypoxic environment, CHD infants are exposed to chronic in utero hypoxia, whereas very preterm infants experience intermittent ex utero hypoxia in addition to other insults, such as infection and inflammation. Genetic variants also play a significant role in CHD pathophysiology, as single mutations can affect multiple organ systems due to shared gene expression pathways in the development of the heart, brain, and placenta (Jin et al., 2017). Thus, CHD-associated mutations may contribute to aberrant development beyond the cardiac abnormality, extending to brain abnormalities that begin prenatally and are consequential for neurodevelopmental outcome in later life (Sadhwani et al., 2022; Wilson et al., 2025). In contrast, the encephalopathy of prematurity is thought to result primarily from prolonged early exposure to the extrauterine environment, disrupting critical neurodevelopmental processes later during gestation. Overall, the fundamental differences in brain structure between these patient groups are likely to be shaped by a complex interplay of different perinatal factors.

While this study advances comparative understanding of early brain structure in high-risk infants, several limitations warrant consideration and point to opportunities for future work. The use of a 40-week atlas for registration may introduce alignment bias in structurally immature brains. While this approach facilitates group comparisons, there may be subtle biases introduced with this method. Neurodevelopmental outcomes were assessed between 18 and 24 months of age, providing an early snapshot of functional consequences. Longer-term follow-up into childhood can offer a more reliable assessment of how early structural alterations relate to later cognitive, motor, and behavioural outcomes, once these domains are more fully developed. Additionally, in infants with CHD, future application of this ICA-based framework to pre- and postsurgical MRI data could help disentangle the contributions of perioperative events to brain development and their combined effects on long-term neurodevelopmental outcome. Together, these future avenues of investigation will advance understanding of the mechanisms linking early brain structure with later neurodevelopment in high-risk infant populations.

In conclusion, the approach taken in this work reflects an entirely data-driven, sensitive detection of aberrant morphometries in CHD and preterm infants, allowing us to compare and contrast neonatal brain structure between these high risk groups. Distinct morphometry typified each group, with spatially distinct networks independently affected by early preterm birth, late preterm birth, and CHD. While both groups commonly present with neonatal brain injury and neurodevelopmental challenges, there may be different antecedent factors driving distinct pathophysiological mechanisms at the cellular and network level, culminating in unique brain regions affected by each pathology.

## Supplementary Material

Supplementary Material

## Data Availability

The control and preterm MRI and neurodevelopmental outcome data are open-access and available at https://biomedia.github.io/dHCP-release-notes/, the MRI and outcome measures for infants with CHD can be made available upon reasonable request with the corresponding author.
