# Supplementary material for: Contrasting neonatal brain morphometry and its impact on neurodevelopmental outcome between preterm birth and congenital heart disease
Source: Imaging Neurosci (Camb). 2025 Dec 17;3:IMAG.a.1063. doi: 10.1162/IMAG.a.1063 (PMC12713110; doi:10.1162/IMAG.a.1063)

Supplementary Table 1. Inclusion and exclusion criteria for MRI data in each group

**dHCP Term**	
**Inclusion criteria**	
≥37 weeks GA at birth	583
**Exclusion criteria**	
major lesion	19
abnormal outcomes (<70 Bayley III Motor/Cognitive)	22
large ventricles	3
reconstruction artefact	2
poor quality images	11
ventriculomegaly	4
RAA/DAA	1
scanner patch < 8	21
syndactly	1
no outcomes	108
incomplete T2 reconstruction	4
1st degree relative with major depressive disorder, autism, or ADHD	25
GMFCS > 0	2
**Total exclusions**	223
**dHCP Term included**	360
**CHD**	
**Inclusion criteria**	
Critical or severe CHD with pre-surgery MRI	
≥37 weeks GA at birth	153
**Exclusion criteria**	
genetic abnormality	4
major lesion	8
<37 weeks PMA at scan	18
missing data	1
CHD type not critical/serious	5
PMA at scan > 46 weeks	1
**Total exclusions**	37
**CHD included**	116
**dHCP Early Preterm**	
**Inclusion criteria**	
≤ 32 weeks GA at birth	92
**Exclusion criteria**	
major lesion	6
poor quality data	2
<37 weeks GA at scan	24
**Total exclusions**	32
**Early preterm included**	60
**dHCP Late Preterm**	
**Inclusion criteria**	
> 32 weeks & < 37 weeks GA at birth	113
**Exclusion criteria**	
poor quality data	4
major lesion	5
<37 weeks GA at scan	37
**Total exclusions**	46
**Late preterm included**	67

Supplementary Table 2. Cohort characteristics for Follow-up Assessment of Neurodevelopmental Outcome, measured using the BSID

Variable	Control (n = 303)	Early Preterm (n = 53)	CHD (n = 72)	p	Post-hoc comparison
Age at Assessment (months), Mean (IQR) *corrected for GA at birth.	19.03 (18.1 – 19.2)	19.4 (18.2 – 19.3)	23.7 (22.1 – 23.9)	< 0.0001	CHD > Control, Early Preterm(*)
Sex (Male)	n = 155 (51%)	n = 31 (58%)	n = 35 (49%)	0.61	-
Index of Multiple Deprivation (IMD) Quintiles. (1 = Most Deprived)	1 = 47<br>2 = 124<br>3 = 61<br>4 = 28<br>5 = 42	1 = 5<br>2 = 17<br>3 =17<br>4 = 3<br>5 = 11	1 = 10<br>2 = 14<br>3 = 13<br>4 = 14<br>5 = 19	0.0006 (**)	CHD > Control, Early Preterm(*)
Cognitive Score, Mean (SD)	102 (10.5)	95 (11.5)	94 (10.6)	8.46e-09 (***)	Control > Early Preterm, CHD(***)
Motor Score, Mean (SD)	104 (8.8)	96 (10.1)	97 (10.2)	2.62e-10 (***)	Control > Early Preterm, CHD (***)

Supplementary Figure 1. Cohort characteristics for Follow-up Assessment of Neurodevelopmental Outcome, measured using the Bayley III

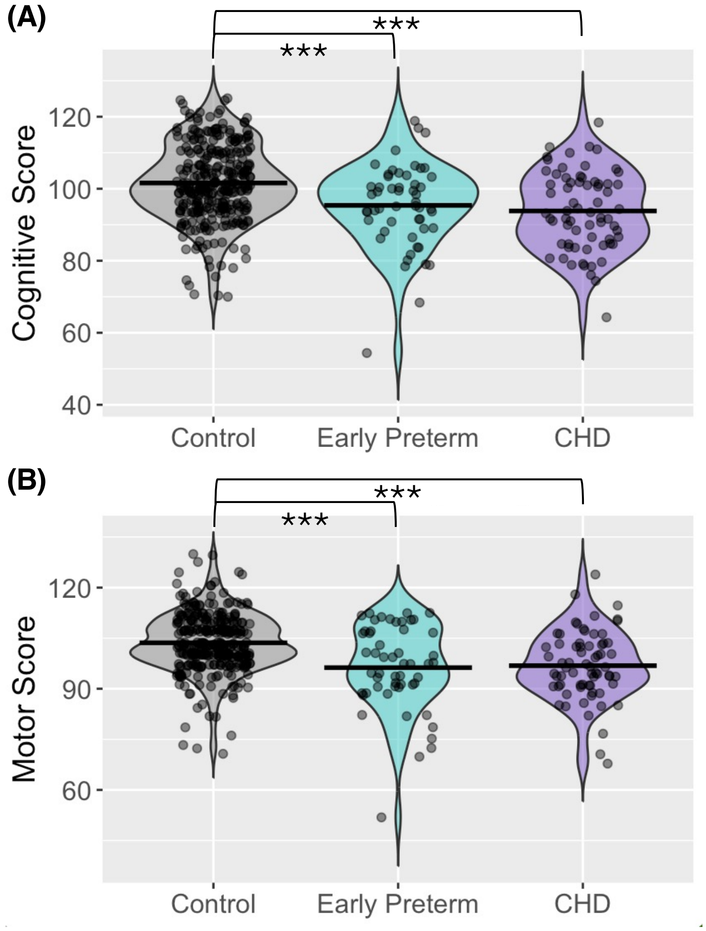

Supplementary Figure 2. Examples illustrating that increasing number of components from n = 40 to n = 50 reduces interpretability of neuroanatomy

(a) Spurious regions that are less precisely neuroanatomical

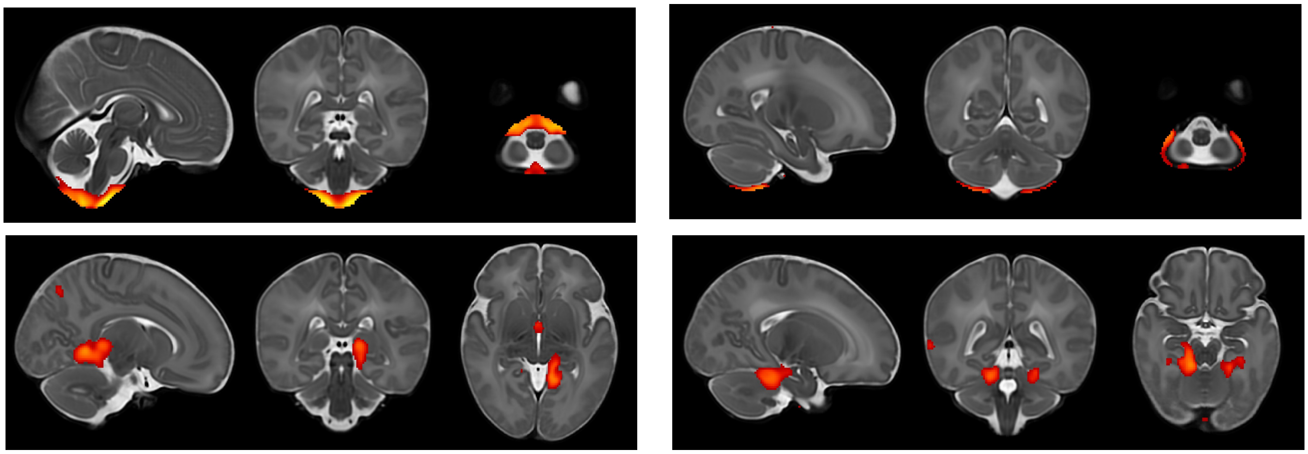

(b) Motor cortex component is smaller and unilateral

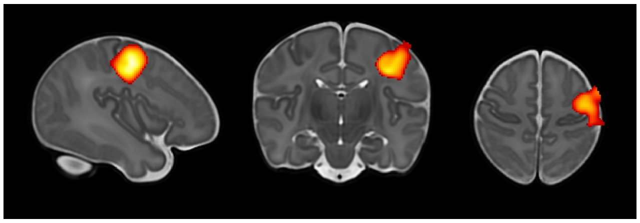

(c) Anterior frontal component becomes unilateral

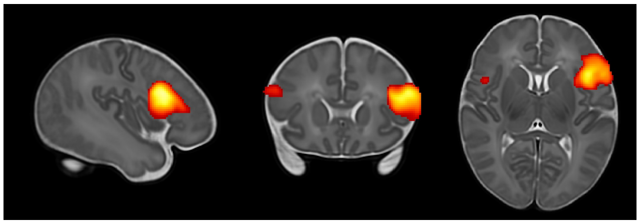

Supplementary Figure 3. ICA applied to CHD Only (n = 40 networks)

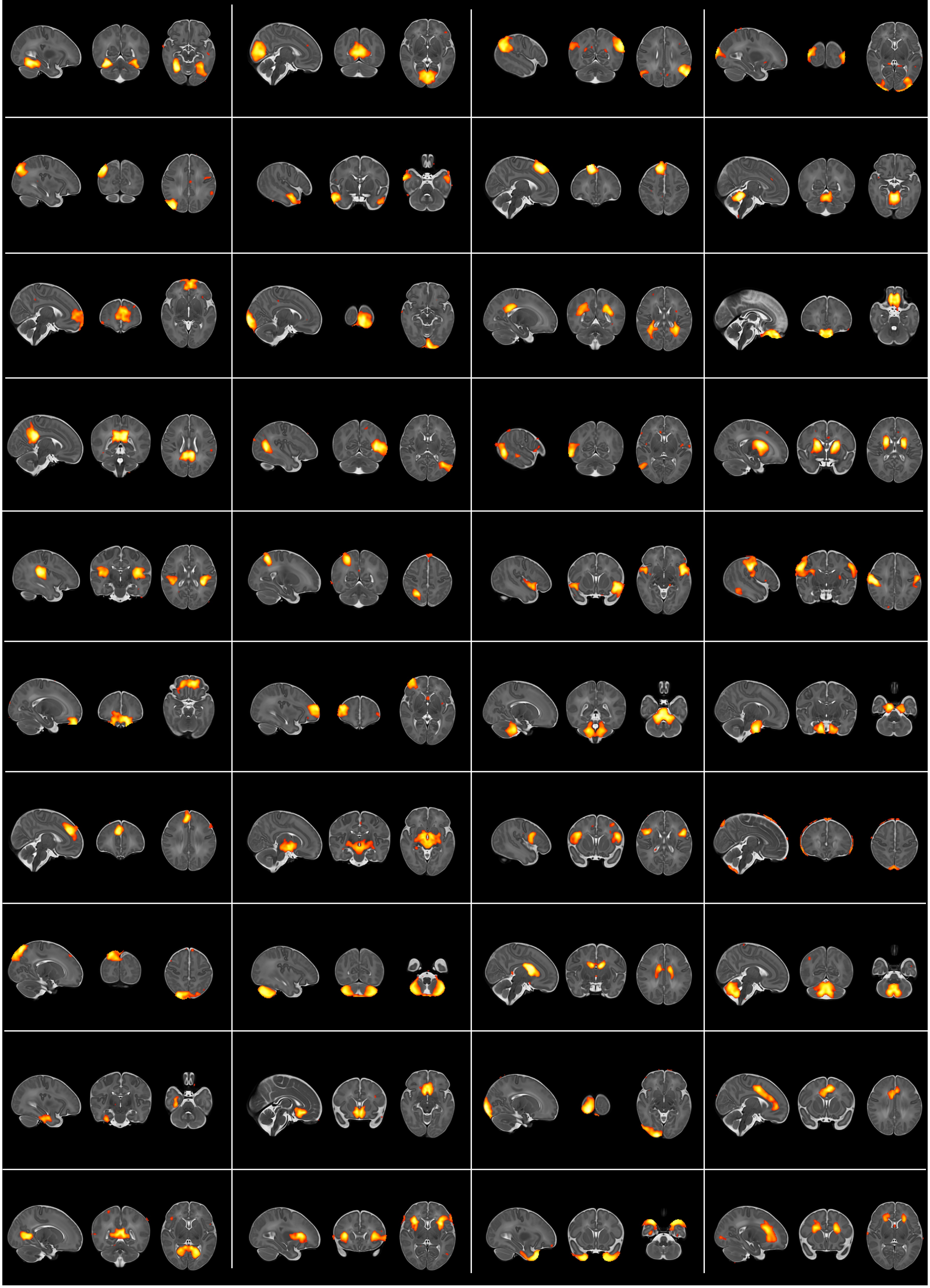

Supplementary Figure 4. ICA applied to Preterm Only (n = 40 networks)

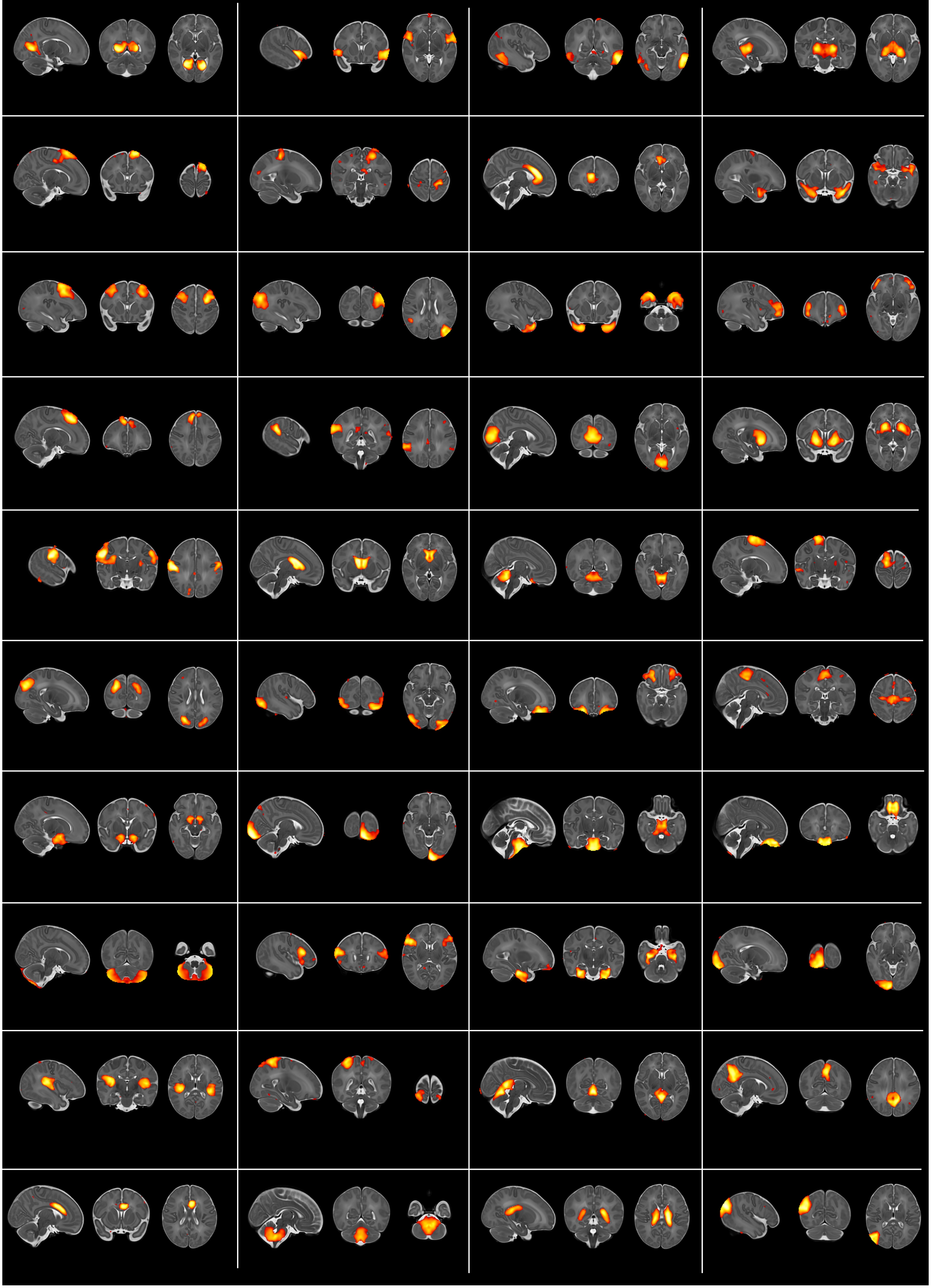

Supplement: Supplementary Material [file IMAG.a.1063_supp.pdf]
